# Prevalence and Risk of Birth Defects Observed in a Prospective Cohort Study: The Hokkaido Study on Environment and Children’s Health

**DOI:** 10.2188/jea.JE20160108

**Published:** 2018-03-05

**Authors:** Tomoyuki Hanaoka, Naomi Tamura, Kumiko Ito, Seiko Sasaki, Atsuko Araki, Tamiko Ikeno, Chihiro Miyashita, Sachiko Ito, Hisanori Minakami, Kazutoshi Cho, Toshiaki Endo, Tsuyoshi Baba, Toshinobu Miyamoto, Kazuo Sengoku, Reiko Kishi, other members of the Hokkaido Study on Environment and Children’s Health

**Affiliations:** 1 Hokkaido University Center for Environmental and Health Sciences, Sapporo, Japan; 2 Graduate School of Health Sciences, Hokkaido University, Sapporo, Japan; 3 Graduate School of Medicine, Hokkaido University, Sapporo, Japan; 4 Department of Obstetrics and Gynecology, Hokkaido University Graduate School of Medicine, Sapporo, Japan; 5 Department of Obstetrics and Gynecology, School of Medicine, Sapporo Medical University, Sapporo, Japan; 6 Department of Obstetrics and Gynecology, Asahikawa Medical University, Asahikawa, Japan

**Keywords:** Hokkaido Study on Environment and Children’s Health, prospective studies, cohort studies, birth defects, growth retardation

## Abstract

**Background:**

Prevalence rates of all anomalies classified as birth defects, including those identified before the 22nd gestational week, are limited in published reports, including those from the International Clearinghouse for Birth Defects Surveillance and Research (ICBDSR). In our birth cohort study, we collected the data for all birth defects after 12 weeks of gestation.

**Methods:**

Subjects in this study comprised 19,244 pregnant women who visited one of 37 associated hospitals in the Hokkaido Prefecture from 2003 through 2012, and completed follow-up. All birth defects after 12 weeks of gestation, including 55 marker anomalies associated with environmental chemical exposures, were recorded. We examined parental risk factors for birth defects and the association between birth defects and risk of growth retardation.

**Results:**

Prevalence of all birth defects was 18.9/1,000 births. The proportion of patients with birth defects delivered between 12 and 21 weeks of gestation was approximately one-tenth of all patients with birth defects. Among those with congenital malformation of the nerve system, 39% were delivered before 22 weeks of gestation. All patients with anencephaly and encephalocele were delivered before 22 weeks of gestation. We observed different patterns of parental risk factors between birth defect cases included in ISBDSR and cases not included. Cases included in ISBDSR were associated with an increased risk of preterm birth. Cases not included in ISBDSR were associated with an increased risk of being small for gestational age at term.

**Conclusions:**

Data from our study complemented the data from ICBDSR. We recommend that birth defects not included in ICBDSR also be analyzed to elucidate the etiology of birth defects.

## INTRODUCTION

Birth defects, including malformations, deformations, and chromosomal abnormalities, are major causes of neonatal mortality.^1^^,^^2^ Previously, it was believed that most birth defects were idiopathic. However, it is now recognized that there are birth defects known to be caused by hazardous epidemics, such as thalidomide exposure during pregnancy. To investigate and prevent birth defects, surveillance programs affiliated with the International Clearinghouse for Birth Defects Surveillance and Research (ICBDSR) are underway.^3^^,^^4^

Incidence of birth defects cannot be accurately estimated because fetal death cases before diagnosis of the pregnancy are unknown. The Japan Association of Obstetricians and Gynaecologists (JAOG) reports observed birth defect cases via the nation-wide hospital-based monitoring program to the ICBDSR. However, mortality cases before 22 weeks of gestation have not been reported.^3^ Data regarding the prevalence of all birth defects, and cases observed before 22 weeks of gestation, could be captured via prospective cohort studies of pregnant women. In this report, we described birth defects observed beginning at 12 weeks of gestation during the pre-natal care of pregnant women in a prefecture-wide hospital-based birth cohort study, the Hokkaido Study on Environmental and Children’s Health.^5^^,^^6^ Furthermore, we examined parental risk factors for birth defects, and the association between the birth defects and the risk of growth retardation. We analyzed and presented the differences in these estimations between those birth defect cases included in the ICBDSR and those cases not included.

## METHODS

### Study cohort

The primary goal of the Hokkaido Study on Environmental and Children’s Health was to examine the effects of perinatal environmental chemical exposures on birth outcomes, including birth defects. The details of this cohort study have been described previously.^5^^,^^6^ We enrolled women in early pregnancy (<13 weeks gestational age), who visited one of the 37 associated hospitals or clinics (including 3 university hospitals and their associated clinics) in the Hokkaido Prefecture, from February 2003 through March 2012. These hospitals and clinics are evenly distributed throughout Hokkaido Prefecture. We obtained written informed consent from all subjects. The institutional ethics board of the Hokkaido University Center for Environmental and Health Sciences (reference no. 14, March 22, 2012) and the Hokkaido University Graduate School of Medicine (May 31, 2003) approved the study protocol.

### Follow-up

Follow-up with the pregnant women enrolled in the study and their offspring is on-going. In this report, we used the dataset of the fixed cohort as of the end of 2015, which included 20,805 women. The number of study participants with a birth record was 19,579. The follow-up rate at birth was 94.1%. Data from 5.9% of participants were missing because the participants were lost to follow-up.

### Data collection

The number of subjects in this report who had birth outcome data and gestational week data was 19,244. According to the standardized manual provided by the principal investigator of the Hokkaido University (R.K.), each physician in charge of each woman in the delivery units of the participating hospitals or clinics filled the unified sheet by referring to the medical records, within 7 days of delivery or at the termination of pregnancy. Whether the diagnosis of birth defects was made during the antenatal period (using ultrasound or via some other means) or during the postnatal period was recorded. However, the date of diagnosis was not recorded. The physicians selected from a list of 55 disease names to record the birth defect, or if the disease was not on the list, described disease names in the unified sheet. These 55 birth defects listed on the unified sheet are possible effect markers of environmental exposure. We encoded the birth defects according to the International Statistical Classification of Diseases and Related Health Problems (ICD), 10th revision.^7^ The ICBDSR monitoring list, which physicians also complete, lists 35 malformations.^3^

Medical records of the parents and offspring at delivery or termination, including gestational age and birth weight, were also recorded on the same sheet. A miscarriage was defined as the presence of a dead fetus between 12 and 21 weeks of gestational age. A stillbirth was defined as the birth of a dead fetus at 22 weeks of gestation or later. Preterm birth was defined as birth between 22 and 36 weeks of gestation. Very low birth weight (VLBW) was defined as birth weight <1,500 g. Small for gestational age at term (term SGA) was defined as birth weight below the 10th percentile reference point for birth weight, according to gestational age, sex, and parity. We used the database of birth weight published by the Japan Pediatric Society as a reference.^8^

The baseline data regarding information on parental reproductive history and lifestyle factors, including age at the entry of this study, body mass index before the pregnancy, parity, drinking habit in the first trimester, smoking during the pregnancy, and any usage of assisted reproductive technologies, were collected using a self-administered questionnaire.

### Statistical analysis

Differences between expected and observed frequencies by gestational week (before week 22 or from week 22 of gestation), sex (males or females), and the number of births (singletons or multiples) for each category or defect were tested using the Fisher’s exact test.

We calculated risk ratios (RRs) for all kinds of birth defects, and birth defects included or not included in the ICBDSR, in singleton fetus or infants, according to maternal and paternal factors, including maternal age at entry (<35 or ≥35 years old), maternal body mass index, parity (0 or ≥1), assisted reproductive technology (used or unused), age of the partner at the entry (<35 or ≥35 years old), maternal alcohol use in early period of the pregnancy (used or unused), and maternal smoking during pregnancy (smoking or nonsmoking). We estimated RRs of birth defects by preterm birth, VLBW, and term SGA. We calculated RRs using log-binomial regression analysis, with and without adjustment for the above maternal and paternal factors. *P* values <0.05 were considered as statistically significant. Statistical analyses were calculated using Stata 14 (Stata Corp, College Station, TX, USA).

## RESULTS

**Figure 1.**
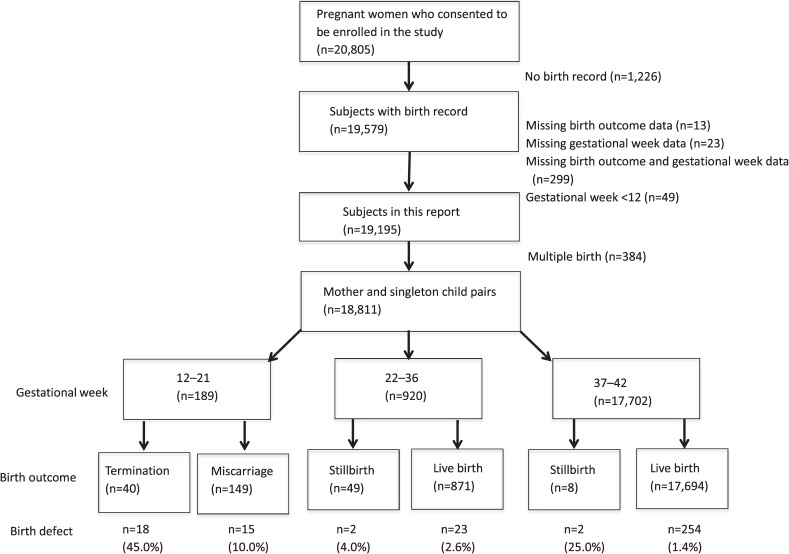
Subjects in this report and the distribution of birth defects according to the gestational week and pregnancy outcomes

We show the distribution of mother and singleton child pairs according to gestational week and birth outcomes in Figure 1. Women who delivered between 12 and 21 weeks of gestation accounted for 10.0% of all births. The proportion of patients with birth defects delivered between 12 and 21 weeks was 9.4% (32/341) of all patients with birth defects observed in this study. Consequently, the prevalence of birth defects among patients delivered between 12 and 21 weeks was approximately 10 times as high as the prevalence of birth defects among patients delivered at 22 weeks of gestation or later. Among study subjects, 40 cases ended in termination and 18 of the 40 cases had a birth defect. Of 149 cases of miscarriage among study subjects, 15 of the cases had a birth defect and of 57 stillbirths, 4 had a birth defect. Of the 18,565 cases that were live born, 277 had a birth defect.

The prevalence of birth defects classified by major ICD-10 categories according to gestational week, sex, and number of births is shown in Table 1. Each defect was counted separately, even if there were accompanying defects in the same infant. The prevalence of all birth defects observed in this study was 18.9/1,000 births (19.7/1,000 pregnant women). The highest prevalence was observed in malformations or deformations of the musculoskeletal system (4.1/1,000 births), followed by malformations of the circulatory system (3.6/1,000 births). The prevalence of birth defects from 22 weeks of gestation was 17.4/1,000 births. The prevalence before 22 weeks of gestation was 164.2/1,000 births (*P* < 0.0001). Prevalence of malformations of the nervous system; malformations of the eye, ear, face, or neck; malformations of the urinary system; malformations and deformations of the musculoskeletal system’ and chromosomal abnormalities was higher before 22 weeks of gestation compared to at 22 weeks of gestation or later. Among patients with congenital malformation of the nerve system, 39% were delivered before 22 weeks of gestation. The total prevalence was not significantly different between males and females: 19.6/1,000 births in males and 17.6/1,000 births in females (*P* = 0.48). Malformations of the eye, ear, face, or neck and malformations of the circulatory system were found more in females than males, but the differences were not statistically significant (*P* = 0.07 and *P* = 0.18, respectively). Malformations of genital organs and the urinary system occurred significantly more in males than females (*P* < 0.001 and *P* = 0.003, respectively). The total prevalence was not significantly different between singleton (18.9/1,000 births) and multiple birth infants (20.8/1,000) (*P* = 0.70). In multiple births, triplet births occurred only in nine pregnancies. No birth defects were observed in the triplet births. Most birth defect cases were identified before birth. All cases of malformation of the nervous system, malformations of the digestive system except for the oral cavity, and malformations of the genital organs were identified before birth. Malformations of the respiratory system showed the lowest percentage of identification before birth (50.0%).

**Table 1.** Prevalence of birth defects by major ICD-10 categories according to gestational age, sex, and multiple birth observed after 12th gestational week in the Hokkaido Study on Environment and Children’s Health^a^

Classification (ICD-10 code)	Total		Gestational week				Sex				Multiple birth				Ascertainment before birth
		
	(*n* = 19,195)		12–21 week		22–42 week		males		females		singleton births		multiple births		(*n* = 19,195)
			(*n* = 201)		(*n* = 18,994)		(*n* = 9,660)		(*n* = 9,437)		(*n* = 18,811)		(*n* = 384)		
	*n*	(/1,000 births)	*n*	(/1,000 births ≤21 weeks)	*n*	(/1,000 births ≥22 weeks)	*n*	(/1,000 male births)	*n*	(/1,000 female births)	*n*	(/1,000 singleton births)	*n*	(/1,000 multiple births)	(%)
Congenital malformations of the nervous system (Q00–Q07)	18	(0.9)	7	(34.8)	11	(0.6)	7	(0.7)	8	(0.9)	18	(1.0)	0	(0.0)	100
Congenital malformations of eye, ear, face, and neck (Q10–Q18)	30	(1.6)	3	(14.9)	27	(1.4)	10	(1.0)	20	(2.1)	30	(1.6)	0	(0.0)	73.3
Congenital malformations of the circulatory system (Q20–Q28)	69	(3.6)	0	(0.0)	69	(3.6)	29	(3.0)	40	(4.2)	68	(3.6)	1	(2.6)	85.5
Congenital malformations of the respiratory system (Q30–Q34)	2	(0.1)	0	(0.0)	2	(0.1)	1	(0.1)	1	(0.1)	2	(0.1)	0	(0.0)	50.0
Cleft lip and cleft palate (Q35–Q37)	36	(1.9)	0	(0.0)	36	(1.9)	19	(2.0)	17	(1.8)	35	(1.9)	1	(2.6)	88.9
Other congenital malformations of the digestive system (Q38–Q45)	19	(1.0)	0	(0.0)	19	(1.0)	12	(1.2)	7	(0.7)	18	(1.0)	1	(2.6)	100
Congenital malformations of genital organs (Q50–Q56)	24	(1.3)	0	(0.0)	24	(1.3)	21	(2.2)	3	(0.3)	23	(1.2)	1	(2.6)	100
Congenital malformations of the urinary system (Q60–Q64)	26	(1.4)	2	(10.0)	24	(1.3)	21	(2.2)	5	(0.5)	22	(1.2)	4	(10.4)	96.2
Congenital malformations and deformations of the musculoskeletal system (Q65–Q79)	79	(4.1)	9	(44.8)	70	(3.7)	43	(4.5)	34	(3.6)	79	(4.2)	0	(0.0)	88.6
Other congenital malformations (Q80–Q89)	28	(1.5)	1	(5.0)	27	(1.4)	12	(1.2)	16	(1.7)	28	(1.5)	0	(0.0)	85.7
Chromosomal abnormalities, not elsewhere classified (Q90–Q99)	32	(1.7)	11	(54.7)	21	(1.1)	14	(1.5)	15	(1.6)	32	(1.7)	0	(0.0)	90.6
Total	363	(18.9)	33	(164.2)	330	(17.4)	189	(19.6)	166	(17.6)	355	(18.9)	8	(20.8)	

ICD, International Statistical Classification of Diseases and Related Health Problems 10th revision.

^a^Each defect was counted separately, even if there were accompanying defects in the same infant.

There were 32 cases of multiple defects. The most frequent combination of multiple defects was malformations of the circulatory system and chromosomal abnormalities (*n* = 8), followed by malformations of the circulatory system and other malformations (*n* = 5) and cleft lip/cleft palate and malformations and deformations of the musculoskeletal system (*n* = 5).

The prevalence of selective birth defects included in the ICBDSR is shown in Table 2. The prevalence of birth defects included in the ICBDSR was 8.4/1,000 births. Cleft lip with or without cleft palate showed the highest prevalence (1.3/1,000 births), followed by Down syndrome (1.0/1,000 births) and polydactyly (1.0/1,000 births). The prevalence of birth defects from 22 weeks of gestation was 7.8/1,000 births. The prevalence before 22 weeks of gestation was 64.7/1,000 births. All patients with anencephaly and encephalocele were delivered before 22 weeks of gestation. Among those with spina bifida, 33% were delivered before 22 weeks of gestation. Most cases were identified before birth. Limb reduction defects showed the lowest percentage of identification before birth (75.0%).

**Table 2.** Prevalence of selected birth defects included in the ICBDSR surveillance program according to gestational age, observed after 12th gestational age in the Hokkaido Study on Environment and Children’s Health^a^

Birth defects	ICD-10 code	Total		Gestational week				Ascertainment before birth

		(*n* = 19,195)		12–21 week		22–42 week		(*n* = 19,195)
				(*n* = 201)		(*n* = 18,994)		
		*n*	(/10,000 births)	*n*	(/10,000 births <22 weeks)	*n*	(/10,000 births ≥22 weeks)	(%)
Anencephaly	Q00	4	(2.1)	4	(20.0)	0	(0.0)	100
Spina bifida	Q05	3	(1.6)	1	(49.8)	2	(1.1)	100
Encephalocele	Q01	1	(0.5)	1	(49.8)	0	(0.5)	100
Microcephaly	Q02	1	(0.5)	0	(0.0)	1	(0.5)	100
Holoprosencephaly	Q04.2	2	(1.0)	0	(0.0)	2	(1.1)	100
Hydrocephaly	Q03	2	(1.0)	0	(0.0)	2	(1.1)	100
Anophthalmos/microphthalmos	Q11.0–Q11.2	0	(0.0)	0	(0.0)	0	(0.0)	
Anotia/microtia	Q16.0, Q16.1	2	(1.0)	0	(0.0)	2	(1.1)	100
Transposition of great vessels	Q20.1–Q20.3	6	(3.1)	0	(0.0)	6	(3.2)	100
Tetralogy of Fallot	Q21.3	5	(2.6)	0	(0.0)	5	(2.6)	60
Hypoplastic left heart syndrome	Q23.4	2	(1.0)	0	(0.0)	2	(1.1)	100
Coarctation of the aorta	Q25.1	3	(1.6)	0	(0.0)	3	(1.6)	100
Choanal atresia, bilateral	Q30.0	0	(0.0)	0	(0.0)	0	(0.0)	
Cleft palate without cleft lip	Q35	11	(5.7)	0	(0.0)	11	(5.8)	81.8
Cleft lip with or without cleft palate	Q36, Q37	25	(13.0)	0	(0.0)	25	(13.2)	92
Oesophageal atresia/stenosis	Q39.0–Q39.4	2	(1.0)	0	(0.0)	2	(1.1)	100
Small intestine atresia/stenosis	Q41	7	(3.6)	0	(0.0)	7	(3.7)	100
Anorectal atresia/stenosis	Q42	6	(3.1)	0	(0.0)	6	(3.2)	100
Undescended testicles	Q53	14	(7.3)	0	(0.0)	14	(7.4)	100
Hypospadias	Q54	8	(4.2)	0	(0.0)	8	(4.2)	100
Indeterminate sex	Q56.4	1	(0.5)	0	(0.0)	1	(0.5)	100
Renal agenesis	Q60	0	(0.0)	0	(0.0)	0	(0.0)	
Cystic kidney	Q61.1–Q61.3	2	(1.0)	0	(0.0)	2	(1.1)	100
Epispadias	Q64.0	0	(1.0)	0	(0.0)	0	(1.0)	
Bladder exstrophy	Q64.1	1	(0.5)	0	(0.0)	1	(0.5)	100
Polydactyly, preaxial	Q69	20	(10.4)	1	(49.8)	19	(10.0)	90
Limb reduction defects	Q71, Q72, Q73	4	(2.1)	1	(49.8)	3	(1.6)	75
Diaphragmatic hernia	Q79.0–Q79.1	5	(2.6)	0	(0.0)	5	(2.6)	100
Omphalocele	Q79.2	0	(0.0)	0	(0.0)	0	(0.0)	
Gastroschisis	Q79.3	0	(0.0)	0	(0.0)	0	(0.0)	
Prune belly sequence	Q79.4	0	(0.0)	0	(0.0)	0	(0.0)	
Trisomy 13	Q91.4–Q91.7	1	(0.5)	0	(0.0)	1	(0.5)	100
Trisomy 18	Q91.0–Q91.3	4	(2.1)	1	(49.8)	3	(1.6)	100
Down syndrome	Q90	20	(10.4)	4	(20.0)	16	(8.4)	90
Total		162	(84.4)	13	(646.8)	149	(78.4)	

ICBDSR, International Clearinghouse for Birth Defects Surveillance and Research; ICD, International Statistical Classification of Diseases and Related Health Problems, 10th Revision.

^a^Each defect was counted separately, even if there were accompanying defects in the same foetus.

RRs of birth defects in singletons for selective maternal and paternal factors are shown in Table 3. For those birth defects included in the ICBDSR, maternal age ≥35 significantly increased birth defect risk (adjusted RR 1.89; 95% CI, 1.23–2.91). For birth defects not included in the ICBDSR, nulliparous and assisted reproductive technology significantly increased birth defect risk (adjusted RR 1.63; 95% CI, 1.13–2.32 and adjusted RR 1.99; 95% CI, 1.06–1.41, respectively). Body mass index, age of partner, alcohol use, and smoking did not significantly increase birth defect risk.

**Table 3.** Risk ratios of birth defects in singleton infants according to maternal factors, observed in the Hokkaido Study on Environment and Children’s Health

	Risk for all birth defects				Risk for Birth defects included in the ICBDSR program				Risk for birth defects not included in the ICBDSR program			
		
	without birth defects	with birth defects	Crude RR (95% CI)	Adjusted RR^a^ (95% CI)	without birth defects^b^	with birth defects	Crude RR (95% CI)	Adjusted RR^b^ (95% CI)	without birth defects^b^	with birth defects	Crude RR (95% CI)	Adjusted RR^b^ (95% CI)
Age at entry												
<35 years old	15,196	243	1.00		15,195	106	1.00		15,195	138	1.00	
≥35 years old	3,301	71	1.34 (1.03, 1.74)	1.61 (1.19, 2.19)	3,301	38	1.64 (1.14, 2.38)	1.89 (1.23, 2.91)	3,301	33	1.10 (0.74, 1.60)	1.40 (0.90, 2.16)
Body mass index												
≥18	15,535	239	1.00	1.00	15,535	113	1.00	1.00	15,535	127	1.00	1.00
<18	1,905	33	1.12 (0.78, 1.61)	1.21 (0.82, 1.778)	1,905	11	0.80 (0.43, 1.47)	0.83 (0.42, 1.65)	1,905	22	1.41 (0.89, 2.20)	1.52 (0.94, 2.45)
Parity												
≥1	11,402	191	1.00	1.00	11,401	98	1.00	1.00	11,401	94	1.00	1.00
0	7,095	123	1.03 (0.83, 1.29)	1.23 (0.94, 1.60)	7,095	46	0.76 (0.53, 1.07)	0.86 (0.57, 1.30)	7,095	77	1.31 (0.97, 1.77)	1.63 (1.13, 2.32)
Assisted reproductive technologies												
No	16,972	254	1.00	1.00	16,971	116	1.00	1.00	16,971	139	1.00	1.00
Yes	743	21	1.86 (1.20, 2.89)	1.95 (1.23, 3.10)	743	9	1.76 (0.90, 3.46)	1.96 (0.97, 3.93)	743	12	1.96 (1.09, 3.51)	1.99 (1.06, 1.41)
Age of the partner												
<35 years old	12,302	192	1.00	1.00	12,302	82	1.00	1.00	12,302	110	1.00	1.00
≥35 years old	6,194	122	1.26 (1.00. 1.57)	1.09 (0.83, 1.43)	6,194	62	1.50 (1.08, 2.08)	1.26 (0.84, 1.87)	6,194	61	1.10 (0.81, 1.50)	0.97 (0.67, 1.89)
Alcohol use in early period of the pregnancy												
No	15,246	228	1.00	1.00	15,245	104	1.00	1.00	15,245	125	1.00	1.00
Yes	2,141	38	1.18 (0.84, 1.66)	1.14 (0.80, 1.66)	2,141	17	1.16 (0.70, 1.94)	1.14 (0.65, 2.01)	2,141	21	1.19 (0.75, 1.89)	1.15 (0.70, 1.89)
Smoking during pregnancy												
No	12,766	210	1.00	1.00	12,766	98	1.00	1.00	12,766	112	1.00	1.00
Yes	2,078	30	0.88 (0.60, 1.29)	0.99 (0.67, 1.45)	2,078	10	0.63 (0.33, 1.20)	0.69 (0.36, 1.33)	2,078	20	1.10 (0.68, 1.76)	1.26 (0.80, 2.04)

CI, confidence interval; ICBDSR, International Clearinghouse for Birth Defects Surveillance and Research; RR, risk ratio.

^a^Adjusted for maternal age, parity, maternal body mass index, and assisted reproductive technology.

^b^Excluding birth defect cases not listed in the ICBDSR surveillance program.

^c^Excluding birth defect cases listed in the ICBDSR surveillance program.

RRs of growth retardation in singletons with birth defects are shown in Table 4. Presence of a birth defect significantly increased the adjusted RRs of VLBW both for birth defects included and those not included in the ICBDSR. For birth defects included in the ICBDSR, presence of a birth defect significantly increased the adjusted RRs of preterm birth (adjusted RR 2.20; 95% CI, 1.34–3.60). Among birth defects not included in the ICBDSR, significantly increased RRs of term SGA was observed (adjusted RR 2.01; 95% CI, 1.11–3.66). Birth defects presented in Table 3 and Table 4 include those observed before 22 weeks of gestation.

**Table 4.** Risk ratios of birth outcomes in singleton infants according to birth defects, observed in the Hokkaido Study on Environment and Children’s Health

	Risk of birth defects				Risk of birth defects included in the ICBDSR program				Risk of birth defects not included in the ICBDSR program			
		
	without birth defects	with birth defects	Crude RR (95% CI)	Adjusted RR^a^ (95% CI)	without birth defects^b^	with birth defects	Crude RR (95% CI)	Adjusted RR^a^ (95% CI)	without birth defects^c^	with birth defects	Crude RR (95% CI)	Adjusted RR^a^ (95% CI)
Preterm birth												
(−)	17,591	289	1.00		17,590	128	1.00		17,590	162	1.00	
(+)	895	25	1.64 (1.12, 2.40)	1.67 (1.13, 2.48)	895	16	2.29 (1.44, 3.66)	2.20 (1.34, 3.60)	895	9	1.09 (0.57, 2.06)	1.21 (0.64, 2.29)
Very low birth weight												
(−)	18,215	277	1.00		18,214	129	1.00		18,214	149	1.00	
(+)	231	33	8.50 (6.01, 12.0)	9.35 (6.57, 13.3)	231	13	7.31 (4.29, 12.5)	8.16 (4.81, 13.8)	231	20	9.45 (6.14, 14.5)	10.20 (6.59, 15.9)
Term small for gestational age												
(−)	15,924	664	1.00		15,919	97	1.00		15,919	117	1.00	
(+)	213	17	1.85 (1.16, 2.93)	1.91 (1.20, 3.03)	664	7	1.68 (0.82, 3.45)	1.75 (0.86, 3.59)	664	10	1.97 (1.08, 3.58)	2.01 (1.11, 3.66)

CI, confidence interval; ICBDSR, International Clearinghouse for Birth Defects Surveillance and Research; RR, risk ratio.

^a^Adjusted for maternal age, parity, maternal body mass index, and assisted reproductive technology.

^b^Excluding birth defect cases not listed in the ICBDSR surveillance program.

^c^Excluding birth defect cases listed in the ICBDSR surveillance program.

## DISCUSSION

The JAOG system is an important nation-wide monitoring system for assessing incidence and prevalence of birth defects and identifying outbreaks that has been in place for approximately 40 years. However, the system aggregates birth defect cases. It is not a population-based registration system, such as those in Scandinavian countries, but a hospital-based monitoring system. The primary difference between the nation-wide reporting of birth defect cases in the JAOG and the present study is that our study is a prospective birth cohort study, in which various data covering all gestational periods, many parental factors, and other related observations, such as infant development after entry to the cohort, were collected, providing additional research and reporting opportunities. In our study, we identified the prevalence of all birth defects after 12 weeks of gestation among the general population of Japanese women in a prefecture-wide prospective cohort study. Our study included 55 birth defects as possible effect markers of environment exposure. We reported that the characteristics of those birth defects not included in the ICBDSR were different from those included in the ICBDSR.

In our study, we were able to examine the above issues because we obtained informed written consent from all women at the time of notification of their pregnancy, or before 13 weeks of gestation. However, we could not include women who miscarried for any reason or cause before the informed consent was obtained. If lethal defects occurred during conception, or before the subjects’ entry into epidemiological studies or surveillance programs, valid incident cases could not be counted. Because an accurate denominator (ie, the number of fetuses at risk) is unknown, this study omitted observations before 12 weeks of gestation. The ICBDSR surveillance programs omit observations before 22 weeks of gestation. Observation before 22 weeks of gestation are included in this report.

The Japanese data reported in the ICBDSR showed that the prevalence of birth defects (total number of cases among live births, stillbirths, and elective terminations of pregnancy for a fetal anomaly) was 1.6% per year during 2007–2011.^3^ Using the same denominator and numerator, the prevalence of birth defects included in the ICBDSR was found to be 0.8% in our study. The prevalence in our study is lower than that reported in the nation-wide hospital-based monitoring project. One possibility is that the ICBDSR monitoring project consists of core hospitals in each area, such as university hospitals and specified children’s hospitals (eg, the Hokkaido Medical Centers for Child Health and Rehabilitation). High-risk pregnant women might tend to visit such hospitals, and severe birth defect cases are usually transferred to such core hospitals before delivery. Moreover, only 10 institutions participated in the monitoring project in the Hokkaido area. Our 37 associated hospitals or clinics, including 3 university hospitals, were evenly distributed throughout Hokkaido Prefecture and accounted for approximately 40% of the institutes with delivery units in this prefecture.^9^ Therefore, we assume that our study participants represented the population of women in general in the Hokkaido area. Another possibility might be that our participants were relatively healthy pregnant women who had an interest in environment and health in communities.

We found that patients with birth defects delivered before 22 weeks of gestation comprised approximately 10% of all patients with birth defects. However, the proportion of birth defects in this early gestational period was very high. Therefore, this finding confirmed that a large proportion of stillbirths and terminations were caused by birth defects. Pregnancies with major structural defects tend to be terminated. Information on termination of pregnancy is difficult to obtain in general; however, prospective birth cohort studies provide an opportunity to obtain information on termination.

Regarding differences by sex, a population-based study in the United States observed that the overall prevalence of major defects in live births was 3.9% among males and 2.8% among females during 1968 to 1995.^10^ We did not observe significant differences in prevalence between males and females. Higher prevalence of malformations of genital organs and urinary system in males, and malformations of ear, face, and neck in females were consistent with data in the United States. However, we found a difference regarding malformations of the circulatory system, with higher prevalence in females in our study. The mechanisms of a sex-based difference in prevalence are unknown. However, race-based difference in prevalence suggests involvement of differences in susceptibility genes.^11^

Concerning multiple gestations, the total prevalence of birth defects was not different between singleton and multiple-birth infants in this study. However, there were congenital malformations observed only in twins. Additional etiological factors appeared to be a factor in multiple births.^12^ Although the prevalence is low, a study of multiple births would be necessary to elucidate the cause of birth defects.

Our study findings suggest a different pattern of parental risk factors between those birth defects included in the ISBDSR and those not included. Various risk factors for birth defects have been suggested, including environmental exposures.^11^^,^^13^ However, the causes of most birth defects remain unknown. The increased risk from high maternal age in our study was consistent with previous studies.^14^ In previous studies, there was less evidence that high paternal age affected risk.^15^ We observed increased risk due to high age of the partner in birth defects included in the ICBDSR, although the RR was not statistically significant. Increased risk of birth defects not included in the ICBDSR due to usage of assisted reproductive technologies was comparable finding to previous studies.^16^ The risk of alcohol use and smoking has been reported in previous studies; however, we did not observe a significant risk.^17^^,^^18^ Future studies need to further examine parental and environmental factors, including passive smoking,^19^ endocrine disrupting chemicals,^20^ indoor air pollution,^21^ folate,^22^^,^^23^ supplemental vitamins,^24^^–^^26^ and stress.^27^^,^^28^

It was indicated in a previous study that structural birth defects contributed to a substantial proportion of preterm birth.^29^ We observed an increased risk of preterm birth in birth defects included in the ICBDSR. In contrast, we observed an increased risk of term SGA in birth defects not included in the ICBDSR. Both low gestational age at birth and SGA result in low birth weight. However, their risk factors and health effects were different between preterm SGA and term SGA infants, a finding which is consistent with previous studies.^13^^,^^30^^,^^31^ Therefore, our findings might suggest that there were different etiological factors between birth defects included and those not included in the ICBDSR. Our observations of birth defects not included in the ICBDSR also suggest that the same etiology, such as usage of assisted reproductive technologies, might be involved in fetal growth and in birth defects. Because of future morbidity of children associated with growth retardation,^32^^,^^33^ our findings emphasize that prospective birth cohort studies play an important role in the prevention of childhood illness.

Birth defects are rare outcomes. In addition, it is often not possible to conduct prospective studies for the investigation of birth defects. Therefore, researchers usually select a case-control study design, which is appropriate for rare disease outcomes, in order to elucidate the relationship between birth defects and parental and environmental factors. However, in case-control studies, an underlying recall bias of exposure is not avoidable.^11^ Although the rarity of specific anomalies often limits the design of epidemiologic studies, the data from prospective studies are still valuable.

The potential disadvantages of our study data should be considered. The findings concerning the lost-to-follow-up group suggest the existence of ‘bias due to withdrawal’, although the reasons for dropout were speculative. Participants from certain backgrounds might tend to withdraw from this or similar studies. However, the effect of the withdrawal was considered to be small because our follow-up rate was sufficiently high.

Malformations, deformations, and chromosomal abnormalities were previously thought to be idiopathic; therefore, they were frequently termed congenital anomalies. However, more recent research indicates that such abnormalities have been caused in part by parental conditions and environmental factors, such as drug usage and environmental pollution. The term ‘congenital anomalies’ is no longer used as the general term.^13^ In this study, the term ‘birth defects’ was used.

Previously, observation of birth defects began at birth. However, timing of ascertainment has begun earlier as technology advances, especially through the use of ultrasound.^11^ In our study, most birth defects were diagnosed before birth. However, some birth defects, such as malformations of the respiratory system, showed low percentage of ascertainment before birth. We continue to collect data regarding birth defects using a self-administered questionnaire administered at 1, 2, 3, 4, and 7 years after delivery. Because there are birth defects that may not be identified until the later years of follow-up, it is anticipated that the number of birth defect cases will increase over time. Future studies investigating the association of risk factors with birth defects and the long-term impacts of birth defects, using the existing and future data of this cohort study, will provide valuable insights.

In conclusion, we reported the prevalence of birth defects in the general population of Japanese women in our cohort study. Although the monitoring system based on the ICBDSR is an excellent nation-wide monitoring system to survey longitudinal trends, the birth defects not included in the ICBDSR should also be analyzed to elucidate the etiology of birth defects. Prospective studies will contribute to the elucidation of the prevalence and etiology of birth defects using the framework of epidemiology.

## APPENDIX

### Members of the Hokkaido Study on Environment and Children’s Health

The other members of the Hokkaido Study, besides the authors, were as follows: H. Goudarzi, M. Minatoya, S. Kobayashi, K. Yamazaki, S. Nishihara, Y. Ait Bamai, R. Miura, S. Kobayashi, A. Uno, S. Katoh, T. Baba, T. Yila, T. Braimoh, and I. Kashino from the Hokkaido University Center for Environmental and Health Sciences, Sapporo, Japan; S. Nakajima and T. Baba from Sapporo Medical University, Sapporo, Japan; Y, Saijyo, E. Yoshioka, T. Miyamoto of Asahikawa Medical College, Asahikawa; K. Okuyama, from Sapporo City Hospital, Sapporo, Japan; F. Sata of Chuou University, Tokyo, Japan; T. Kita of the Hokkaido Information University, Ebetsu, Japan.
